# Systematic Analysis of the Stress-Induced Genomic Instability of Type Three Secretion System in *Aeromonas salmonicida* subsp. *salmonicida*

**DOI:** 10.3390/microorganisms9010085

**Published:** 2020-12-31

**Authors:** Pierre-Étienne Marcoux, Antony T. Vincent, Marie-Ange Massicotte, Valérie E. Paquet, Émilie J. Doucet, Nava Hosseini, Mélanie V. Trudel, Gabriel Byatt, Mathilde Laurent, Michel Frenette, Steve J. Charette

**Affiliations:** 1 Institut de Biologie Intégrative et des Systèmes, Université Laval, Pavillon Charles-Eugène-Marchand, Quebec City, QC G1V 0A6, Canada; pierre-etienne.marcoux.1@ulaval.ca (P.-É.M.); massicom@mcmaster.ca (M.-A.M.); valerie.paquet@criucpq.ulaval.ca (V.E.P.); emilie.doucet.4@ulaval.ca (É.J.D.); nava.hosseini.1@ulaval.ca (N.H.); melanie.trudel@live.ca (M.V.T.); gabriel.byatt.1@ulaval.ca (G.B.); mathilde.laurent@gmx.com (M.L.); 2 Hôpital Laval, Centre de Recherche de l’Institut Universitaire de Cardiologie et de Pneumologie de Québec, Quebec City, QC G1V 4G5, Canada; 3 Département de Biochimie, de Microbiologie et de Bio-Informatique, Faculté des Sciences et de Génie, Université Laval, Quebec City, QC G1V 0A6, Canada; michel.frenette@bcm.ulaval.ca; 4 Département des Sciences Animales, Faculté des Sciences de L’agriculture et de L’alimentation, Université Laval, Quebec City, QC G1V 0A6, Canada; antony.vincent@fsaa.ulaval.ca; 5 Groupe de Recherche en Écologie Buccale (GREB), Faculté de Médecine Dentaire, Université Laval, Quebec City, QC G1V 0A6, Canada

**Keywords:** type three secretion system, pAsa5, *Aeromonas salmonicida* subsp. *salmonicida*, rearrangement, *AsaGEI*, prophage

## Abstract

The type three secretion system (TTSS) locus of *Aeromonas salmonicida* subsp. *salmonicida*, located on the plasmid pAsa5, is known to be lost when the bacterium is grown at temperatures of 25 °C. The loss of the locus is due to the recombination of the insertion sequences flanking the TTSS region. However, the mechanism involved in this recombination is still elusive. Here, we analyzed 22 *A. salmonicida* subsp. *salmonicida* strains that had already lost their TTSS locus, and we systematically explored another 47 strains for their susceptibility to lose the same locus when grown at 25 °C. It appeared that strains from Europe were more prone to lose their TTSS locus compared to Canadian strains. More specifically, it was not possible to induce TTSS loss in Canadian strains that have *AsaGEI2a*, a genomic island, and prophage 3, or in Canadian strains without a genomic island. A comparative genomic approach revealed an almost perfect correlation between the presence of a cluster of genes, not yet characterized, and the susceptibility of various groups of strains to lose their locus. This cluster of genes encodes putative proteins with DNA binding capacity and phage proteins. This discovery creates new opportunities in the study of pAsa5 thermosensitivity.

## 1. Introduction

The type three secretion system (TTSS) is a virulence factor found in many Gram-negative bacteria and is essential for the virulence of the fish pathogen *Aeromonas salmonicida* subsp. *salmonicida*. This needle-like protein structure allows for the translocation of effectors from the bacteria’s cytosol directly into the host cell. Once in the host cells, the effectors disrupt various cellular functions and thus contribute to the pathogenicity of the bacteria [1].

*A. salmonicida* subsp. *salmonicida* is a psychrophilic bacterium with an optimum growth temperature below 20 °C. The proteins that form the structure of the TTSS are encoded in a region of the large 155 kbp plasmid named pAsa5, also known as pASvirA [2,3]. The pAsa5 plasmid is unstable during growth at a high temperature such as 25 °C, and can lose the region that encodes for the TTSS. Given the high importance of the TTSS in the pathogenicity of the bacterium, the strains that lose their TTSS locus are avirulent [3,4,5,6].

**Figure 1:**
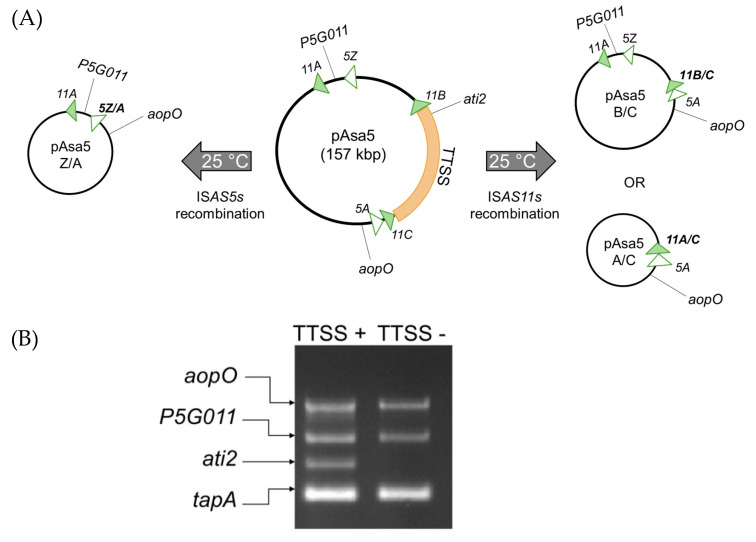
(**A**) Schematic representation of 3 recombination patterns of pAsa5 according to the ISs present on the plasmid. The region in orange corresponds to the TTSS locus, the IS*AS11*s are represented by green triangles and the IS*AS5*s are shown by white triangles. This figure is inspired by Tanaka et al. [8] (**B**) An example of the PCR multiplex result from the SHY14-2246 strain, which contains an unaltered pAsa5 plasmid (TTSS+) and a strain that has lost its TTSS genes (TTSS−) generated from SHY16-3432 parental strain. The *AopO* and *P5G011* genes are located downstream and upstream, respectively, of the region encoding the TTSS. The third gene corresponds to *ati2*, which is located inside the TTSS locus. The last gene (*tapA*) is located in the chromosome and it is used as a positive control.

Though this phenomenon was initially suspected to be due to a complete loss of the pAsa5 plasmid [3], it is, in fact, homologous recombination between insertion sequences (ISs) present on the plasmid that results in the loss of the coding region for the TTSS [7,8]. The recombination between copies of the same ISs induced by growth at high temperatures is not exclusive to *A. salmonicida*. In other species like *Bacillus*, the heat-induced transposition of ISs has already been observed [9] and the use of ISs as a template for recombination was reported for other bacterial species [10]. For the *A. salmonicida* subsp. *salmonicida* A449 reference strain, the rearrangements of pAsa5 that led to the loss of the TTSS were observed between the IS*AS11A* and IS*AS11C* (A/C) or IS*AS11B* and IS*AS11C* (B-C). The pAsa5 plasmid of the 01-B526 strain has an additional IS*AS5* compared to the A449 strain, which can undergo rearrangements via IS*AS5Z* and IS*AS5A* (Z/A), in addition to the A–C and B–C rearrangements [7,8] (Figure 1).

In a previous study, an analysis of three strains showed that they exhibited a different ability to generate rearrangement for the pAsa5. It was possible to produce derivatives that had lost their TTSS locus for the 01-B526 and A449 strains, whereas no clone without the TTSS could be obtained for the 01-B516 strain [4,8]. This result suggests that it is unlikely that all strains lose their TTSS when exposed to stress. As the strains of *A. salmonicida* subsp. *salmonicida* have a rich accessory genome (plasmids, prophages, genomic islands, etc.), which varies from strain to strain, it is possible that one or more of these mobile DNA elements may contribute to the difference in susceptibility between strains to lose their TTSS.

Among the mobile DNA elements found in *A. salmonicida* subsp. *salmonicida*, there are the *Aeromonas salmonicida genomic islands* (*AsaGEI*s) present in several strains of the bacterium. Genomic islands are genetic elements acquired by horizontal transfers and inserted into the chromosome [11]. *AsaGEI*s genes encode for putative prophage proteins, but their role in the bacteria is still unknown. However, they are interesting from an epidemiological point of view because each type of *AsaGEI* is associated with a distinct geographic region [12]. Depending on their sequence and especially their integrase gene, *AsaGEI*s can be divided into 5 types (*1a*, *1b*, *2a*, *2b,* and *2c*) and have a size varying from 50 to 53 kb [12,13,14]. Strains with an *AsaGEI1a* or *2a* are from North America and strains with an *AsaGEI1b* and *2b* are found in Europe. A strain that has an *AsaGEI2c* has been described in China. The *AsaGEI1*s have the same insertion site in the chromosome which is different from the one of the *AsaGEI2*s [12]. Furthermore, *A. salmonicida* subsp. *salmonicida* strains only contain one of the 5 types within their genome at a time and some strains contain no *AsaGEI* at all [12].

Two prophages are found in nearly all strains of *A. salmonicida* subsp. *salmonicida*. These are prophages 1 and 2, which were originally described in the A449 strain [2]. Another prophage, prophage 3, was described later and has so far only been found in North American isolates along with *AsaGEI2a* [12].

To better understand why certain strains of *A. salmonicida* subsp. *salmonicida* are more likely to lose their TTSS locus, we systematically studied the phenomenon in nearly 50 strains from various origins. This study confirms that the strains have a variable propensity to lose their TTSS locus. To further study this phenomenon, we performed a comparative genomic analysis which allowed us to identify a cluster of genes that could be involved in the molecular mechanism underlying the TTSS rearrangement.

## 2. Materials and Methods

### 2.1. Bacterial Strains and Regular Growth Conditions

The *A. salmonicida* subsp. *salmonicida* strains used in this study are described in Table 1 and the Supplementary Materials. They were grown from the frozen stock for three days at 18 °C on furunculosis agar [4].

### 2.2. Production of TTSS-Rearranged Strains

For each studied strain, clones without TTSS were produced by the previously described protocol [4], but with shorter incubation periods. Briefly, each strain was streaked on furunculosis agar from a stock at −80 °C and incubated at 25 °C for 3 days. Subsequently, 5 isolated colonies were subcultured, streaked on furunculosis agar and incubated at 25 °C for 3 additional days. After this incubation, a multiplex PCR analysis (see next section) was performed on 6 isolated colonies per Petri dish for a total of 30 colonies tested for each strain under study.

### 2.3. PCR Multiplex

This method was designed to identify strains that have lost certain genes present on pAsa5. The primers used are listed in Table S1. These primers targeted 3 genes (*P5G011*, *ati2* and *aopO*) located at different positions on the plasmid. The fourth gene targeted is *tapA*, which is a positive control for each bacterial lysate. DNA templates were prepared by lysing one bacterial colony in 20 µL of SWL buffer (50 mM KCl, 10 mM Tris, pH 8.3, 2.5 mM MgCl_2_, 0.45% NP-40, and 0.45% Tween 20) [15]. The lysates were heated at 95 °C for 10 min. The PCR mixture and conditions were the same as previously described [16], except that the four primer pairs were mixed together. The ratio of each primer pair in the mix was: 1,6:1,2:0,6:1 for *aopO*, P5G011, *ati2* and *tapA*, respectively). The PCR program was as follows: 2 min 30 s at 95 °C, 30 cycles of 30 s at 95 °C, 30 s at 60 °C, and 1 min at 68 °C, followed by a final 10 min extension at 68 °C. The samples were separated on 1.3% agarose gels, which were stained with 0.5 µg/mL ethidium bromide.

### 2.4. Comparative Genomics

To learn more about genomic determinants, which may help explain the propensity of certain strains to rearrange their genome, genes unique to strains with an *AsaGEI1a*, *1b,* or, without *AsaGEI* from Europe, were compared to Canadian strains with an *AsaGEI2a* (Table S2). To do this, the genomes were annotated by RASTtk through the PATRIC web server [17]. Subsequently, PATRIC’s Protein Family Sorter was used to define groups of homologous proteins according to the stringent parameter (MCL inflation = 3.0) genus-specific families (PLfams) as recommended by PATRIC for close strain comparisons. The genomic sequences were visualized with Artemis version 18.1.0 [18] and Easyfig version 2.2.2 [19].

### 2.5. PCR Analyses for Genotyping

The PCR primers that were used are listed in Table S1. The procedure is similar to multiplex PCR, except that only one pair of primers was used at a time, and the incubation period at 68 °C was 30 s instead of one minute. For amplicons longer than 3 kbp, the LongAmp Taq (New England BioLabs, Whitby, ON, Canada) was used as previously described [8]. The PCR assays were performed at least twice, and appropriate positive and negative controls were included with each assay.

## 3. Results

In 2011, we demonstrated that many *A. salmonicida* subsp. *salmonicida* strains had abnormalities in their pAsa5 plasmid with the absence of the TTSS locus and other regions [4]. In the years that followed, our team accumulated a large collection of *A. salmonicida* subsp. *salmonicida* and several of these also lacked the TTSS locus on their pAsa5 (Table 1). Throughout additional studies, we have produced strains without their TTSS locus using different heat stress protocols (Table 1). The compilation of all these 22 strains without TTSS locus shows that 8 of these strains came from Europe and do not have *AsaGEI*, 5 rearranged strains were from Quebec and bear *AsaGEI1a*, and 9 other strains without TTSS have various origins.

**Table 1:** Strains of *A. salmonicida* subsp. *salmonicida* which have already lost the genes encoding TTSS before their inclusion in our strain collection or which have lost it in the laboratory following incubation at 25 °C.

Type of *AsaGEI*	Strains	Origin	How TTSS Has Been Lost ^a^	Reference
No *AsaGEI*	HER1098	USA	Already lost	[4]
	HER1110	Japan	Already lost	[4]
	HER1104	France	Already lost	[4]
	HER1084	France	Already lost	[4]
	RS 534	France	Already lost	This study
	RS 887	Russia	Already lost	This study
	JF3519	Switzerland	Already lost	[16]
	JF3791	Switzerland	Already lost	[16]
	A449	France	2 weeks	[4]
	JF2267	Switzerland	Few hours	[3]
*AsaGEI*2a (without prophage 3)	07-5957	Quebec, Canada	Already lost	[4]
	07-7346	Quebec, Canada	3 days	This study
*AsaGEI*2b	JF3224	Switzerland	Already lost	[14]
*AsaGEI*1a	07-7287	Quebec, Canada	Already lost	[4]
	m14349-09	Quebec, Canada	3 days	This study
	RS 1705	Ontario, Canada	3 days	This study
	M10745-12	Quebec, Canada	3 days	This study
	M17930-12	Quebec, Canada	3 days	This study
	01-B526	Quebec, Canada	2 weeks	[4]
*AsaGEI*1b	HER1108	Denmark	Already lost	[4]
	JF2869	Switzerland	Already lost	This study
	HER1085	Norway	3 days	[4]

^a^: This column indicates the culture protocol at 25 °C which was used and which led to the observation of the loss of TTSS. These protocols lasted a few hours, 3 days, or two weeks depending on the case. Several strains had lost their TTSS even before our laboratory acquired them. In these cases, it is not possible to know the culture conditions that led to the loss of the TTSS.

**Figure 2:**
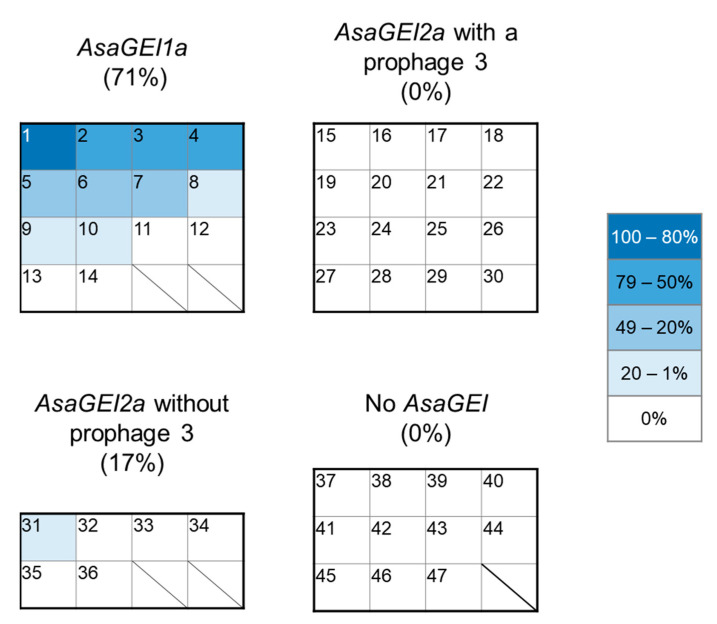
Systematic analysis of the TTSS locus loss in Canadian strains of *A. salmonicida* subsp. *salmonicida* after incubation at 25 °C for two consecutive periods of 3 days. Each numbered square corresponds to a strain. The strains where the TTSS locus loss has been observed are shown in blue. The percentage of rearrangement was calculated by the number of colonies that lost their TTSS out of the colonies tested (see Table S3 for the complete results).

To have a more precise and complete understanding of the phenomenon for the loss of the TTSS by rearrangement of the pAsa5 plasmid and the factors that may influence it, we selected 47 strains from Canada, particularly from the Province of Quebec, to study their ability to lose their TTSS during heat stress. These strains fall into four groups, depending on the type of *AsaGEI* they have or lack, and the presence of prophage 3 (Figure 2). These strains were cultured under stressful conditions at 25 °C for two consecutive periods of 3 days then 30 colonies of each of these strains were analyzed by PCR genotyping to investigate the presence of the TTSS (Figure 1). As shown in Figure 2, 71% of the strains bearing an *AsaGEI1a* had colonies that lost their TTSS. This was the case for only one strain with an *AsaGEI2a* without prophage 3. Strains with an *AsaGEI2a* and a prophage 3 or without an *AsaGEI* did not lose their TTSS at all. However, even within the group of strains that have *AsaGEI1a*, there is great variability in the predisposition of strains losing their TTSS (Figure 2 and Table S3).

Following this systematic heat stress protocol, it does not seem possible to induce TTSS loss in Canadian strains that bear *AsaGEI2a* and prophage 3 and strains without *AsaGEI* from Canada under the tested conditions. However, since we were only testing 30 colonies per strain, we hypothesized that TTSS loss can be lower than 3% (i.e., less than 1 colony on 30 analyzed) for these groups of strains and that more colonies needed to be tested to detect at least one rearranged strain. To this end, all the colonies isolated on different agar plates for the 01-B516 strain, which is a refractory strain with *AsaGEI2a* and prophage 3, were analyzed to test this hypothesis. A total of 967 colonies were tested and none of them lost the TTSS locus based on the presence of the *ati2* gene in all the PCR tests (Table S3). Consequently, based on this result, we proposed a second hypothesis: a genetic element present or absent in susceptible strains must explain the loss of the TTSS locus during heat stress. Since European strains without *AsaGEI* are susceptible to lose their TTSS locus, this genetic element does not appear to be an *AsaGEI.* Therefore, a comparative genomic analysis was mandatory.

Based on the genomic sequences already available for several of the strains included in this study (Table S2), we searched for genetic elements specific to groups of strains that may lose their TTSS locus, or, conversely, elements specific to the strains that cannot lose it. An analysis carried out with the PATRIC web server identified only seven adjacent genes (ASA_2927 to ASA_2933 according to the genome annotation of the reference strain A449) specifically present in strains A449, 01-B522, 01-B526, CIP 103209, 170-68, RS534, JF3791, JF3517, JF2267, and JF2506, which all can lose their TTSS; and absent from the genomes of strains 2004-05MF26 and M22710-11, which cannot lose their TTSS by rearrangement.

**Figure 3:**
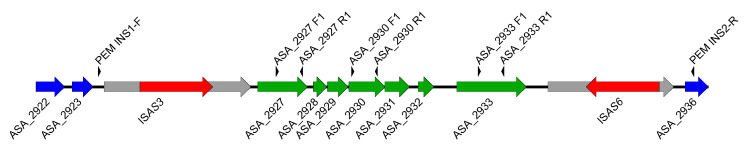
Map of the chromosomal region of the A449 strain where the ASA_2927 to ASA_2933 genes are found. These genes are specific to strains that can lose their TTSS locus. The ASA_2927 through ASA_2933 genes are shown in green. A gene encoding a putative integrase (in gray on the left of the figure) is interrupted by an IS*AS3*, an IS (in red). Another gene (in gray on the right of the figure), coding for a hypothetical protein, is interrupted by IS*AS6*, another IS (in red). The arrowheads above the genes indicate the position of primers used in genotyping PCR.

The chromosomal locus of strain A449, where the ASA-2927 to ASA-2933 genes are located, is shown in Figure 3. These 7 genes are flanked by two genes which are both interrupted by ISs. The first of these genes, upstream of ASA_2927, codes for a putative integrase. The proteins produced by the seven genes have the following putative functions: ASA_2927 = hypothetical protein, ASA_2928 = AlpA family transcriptional regulator, ASA_2929 = helix-turn-helix domain-containing protein, ASA_2930 = ash family protein, ASA_2931 = hypothetical protein, ASA_2932 = hypothetical protein, ASA_2933 = inovirus-type Gp2 protein.

**Figure 4:**
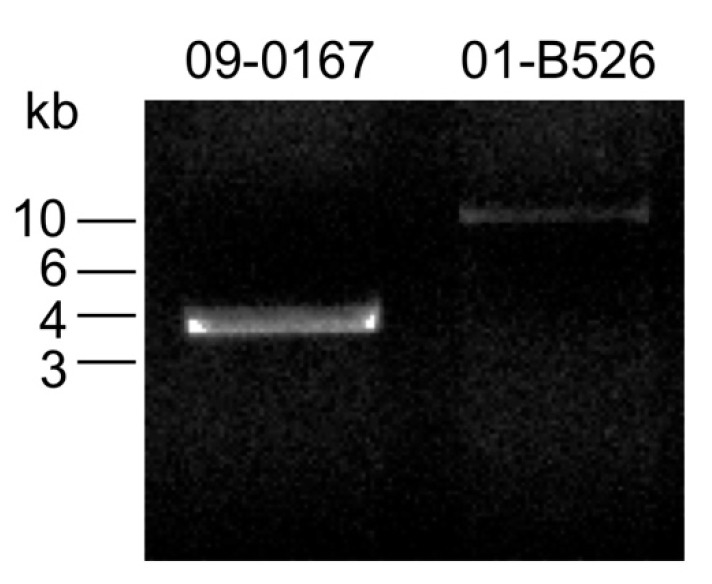
PCR amplification of the region that contains the gene cluster. The primers PEM_INS1-F and PEM_INS2-R were used (see Figure 3 for their position on the chromosome). Based on genomic analysis, the 01-B526 strain contains the gene cluster while the 09-0167 strain does not.

Primers PEM_INS1-F and PEM_INS2-R were designed to analyze the presence of this entire gene cluster by PCR (Figure 3). The primer pair amplifies a 10 kb region in 01-B526, a strain capable of losing its TTSS locus. In the 09-0167 strain, which is unable to lose its TTSS locus, an amplicon of about 3.7 kb is obtained (Figure 4). The difference in size between the amplicons is consistent with the presence of the ASA_2927 to ASA_2933 genes in the 01-B526 strain compared to the 09-0167 strain. By analyzing the genome of the 09-0167 strain (RefSeq assembly accession: GCF_001902165.1), it was possible to determine that the two ISs are still present but the entire segment between the two ISs, except for 13 nucleotides, is not present in this strain. The sequence portion of the putative integrase gene upstream of IS*AS3* and the sequence portion of the last gene downstream of IS*AS6* are present in the 09-0167 genome, which suggests that the ancestor of this strain has lost the genes between the ISs.

Following genomic analysis, the 69 strains of *A. salmonicida* subsp. *salmonicida* from Table 1 and Table S3 were tested to see if they contain the gene cluster by using primer pairs that target genes ASA_2927, ASA_2930, and ASA_2933 (Figure 3). Table 2 shows that the gene cluster is present in all the *AsaGEI1a*-bearing strains and all the strains without *AsaGEI* originating from outside Canada. For the *AsaGEI2a* strains and the Quebec strains without *AsaGEI*, the gene cluster is absent. For strains that do not have the gene cluster, the same 3.7 kb as for the 09-0167 strain is obtained with the PEM_INS1-F and PEM_INS2-R primers (Figure 4). All these results propose a link between TTSS loss and the presence of the gene cluster. The only group of strains where this conclusion does not fit is for the strains bearing an *AsaGEI2a* without prophage 3. Based on Table 1 and Table S3, from the 8 strains of this group that were analyzed, three can lose their TTSS, even if the gene cluster was not detected in their genome.

## 4. Discussion

In this study, we confirmed the previously proposed idea that *A. salmonicida* subsp. *salmonicida* strains are not all susceptible to the loss of their TTSS when exposed to stressful temperatures [4,7,8]. We performed a systematic analysis of 69 strains, while the previous conclusions had been based on analyses of only three strains (A449, 01-B516, and 01-B526). Our in-depth analysis has made it possible to divide strains according to their ability to lose their TTSS locus, and according to the presence of different genetic elements like *AsaGEIs* and prophage 3, which are in part linked to their geographical origin [12].

The majority of the strains that can lose their TTSS are strains with an *AsaGEI1a* (from Quebec, Canada), or strains that come from outside Canada, regardless of the presence of an *AsaGEI*. This study identified two groups of totally refractory strains: strains that have *AsaGEI2a* and prophage 3, as well as strains without *AsaGEI* from the province of Quebec. In these two groups, none of the strains have lost their TTSS locus (Table 2). Despite testing nearly one thousand colonies for 01-B516, no colony without the TTSS has been obtained. This clearly demonstrates that some strains are refractory to the loss of the TTSS with the heat stress protocol used. Moreover, in Daher et al., 2011, we tested strain 01-B516 for 2 weeks instead of 6 days without success for a total of 90 colonies. Therefore, increasing the incubation time may not have the additional impact of losing the TTSS locus [4].

The discovery of a gene cluster within *AsaGEI1a*-bearing strains and in strains from outside Canada is very interesting. The presence of this gene cluster correlates with many of the strains that lose their TTSS, suggesting that this genetic element could be involved in the rearrangement of pAsa5. That said, only 71% of the strains from the susceptible group with an *AsaGEI1a* lose their TTSS locus. Among the sensitive strains, some, such as 07-9324, 08-2783, 08-4188, and M15879-11, had more than 50% of their tested colonies devoid of TTSS locus. By comparison, only one or two colonies without the TTSS locus were found in three strains (01-B522, SHY16-3432, and SHY18-3337, see Table S3).

Consequently, the mechanism behind the recombination of the ISs found on each side of the TTSS locus in pAsa5 is more complex than an on/off process. Regulatory mechanisms are likely included in this process since the loss of the TTSS has major consequences on the virulence of the bacterium [3,4,5,6]. On the other hand, we should also consider that *AsaGEI1a*-bearing strains that did not lose their TTSS may need greater stress to induce the process; another possibility is that the gene cluster they contain that is suspected of being involved in the process may contain mutations that inhibit its action in the recombination of pAsa5. In the present study, our systematic approach was applied on *A. salmonicida* subsp. *salmonicida* strains isolated in Canada only and mainly from the Province of Quebec, because of the availability of these strains in our bacterial collection. Based on the results we obtained for the *AsaGEI1a*-bearing strains, where their sensitivity to lose their TTSS was variable from one strain to the other, we can predict that a similar observation would apply for strains from outside Canada that bear the gene cluster.

*A. salmonicida* subsp. *salmonicida* has a rich plasmidome. Some plasmids, such as the cryptic plasmids pAsa1, pAsa2, and pAsa3, are found in nearly all the strains [16,20]. Other plasmids, especially the antibiotic resistance plasmids, are distributed heterogeneously [21]. Considering the non-uniform distribution of plasmids, we wanted to know if the presence or absence of some plasmids was a factor in the strain’s ability to lose their TTSS.

As shown in Table S4, there is no support for this idea. The pAsa9 plasmid is only present in the first sensitive group (*AsaGEI1a*). The plasmid pSN254b is present in many strains that have *AsaGEI1a,* but it is also in one *AsaGEI2a* strain with prophage 3. These two plasmids are absent from the sensitive strains outside Canada without an *AsaGEI*. Consequently, the presence or absence of pAsa9 and pSN254b plasmids, or indeed any other plasmid, does not correlate with the ability of a strain to lose its TTSS genes.

The loss of the TTSS in some *AsaGEI2a*-bearing strains without prophage 3 compared to strains that have both *AsaGEI2a* and prophage 3 may suggest a negative regulation of the pAsa5 recombination processes by elements encoded by prophage 3. However, it could be another genetic element, not yet identified in *AsaGEI2a*-bearing strains without prophage 3, that could explain their sensitivity to heat stress. Further analysis, including sequencing and analyzing additional genomes, will be required to shed light on this.

The strains which do not have the gene cluster composed of ASA_2927 to ASA_2933 still bear traces of the presence of the latter. This suggests that these strains have evolved to stop losing their TTSS and opens interesting ecological questions for future research. In addition, the molecular mechanism that led to the loss of the ASA_2927 to ASA_2933 genes is puzzling, since it is not possible to explain how this loss of genes could have occurred. The two unrelated ISs ended up aligned with each other. It is therefore possible that these ISs are involved in the process leading to the loss of this gene cluster. However, compared to the loss of the TTSS from pAsa5, the mechanism of which is starting to be clarified, the process behind the loss of the gene cluster remains unknown at this time. It is also interesting to note that the ISs bordering the gene cluster (IS*AS3* and IS*AS6*) are not the same as those known to be able to generate a rearrangement of the TTSS (IS*AS5* and IS*AS11*). However, IS*AS3* and IS*AS6* are of the same family (IS*3*), while ISAS*5* and IS*AS11* are members of the IS*21* and IS*256* families, respectively [2].

## 5. Conclusions

The bacterium *A. salmonicida* subsp. *salmonicida* is the causative agent for furunculosis in salmonids. This disease causes a high mortality rate, which results in heavy economic losses in aquaculture around the world [22]. Generally, the vaccines available in aquaculture use dead bacteria that have been inactivated by different processes [23]. However, the efficacy rate for these vaccines is low for all methods of delivery [24]. Vaccine formulation from live attenuated strains is an attractive alternative for vaccine development and has been studied with live strains of *A. salmonicida* subsp. *salmonicida* whose virulence has been attenuated [25]. A study by Origgi et al. suggested using strains without TTSS as vaccines to develop an immune response [26]. Our study revealed that a large proportion of *A. salmonicida* subsp. *salmonicida* strains can lose their TTSS locus to become vaccine candidates.

As a next step in our study of the TTSS locus instability, it will be of interest to dissect the molecular mechanism that links the newly discovered gene cluster to the TTSS locus lost by the pAsa5 rearrangement driven by the IS recombination. It is also possible that bacterial host mechanisms are also involved in the process and hijacked by the proteins encoded in the gene cluster. However, as suggested by strains bearing the *AsaGEI2a* who lost their TTSS in the absence of the gene cluster, at least another mechanism yet to be discovered can also trigger the rearrangement of pAsa5.

Finally, in addition to studying the exact role of the gene cluster in TTSS locus loss, it will be interesting to determine the spread of this genetic element in the genome of *A. salmonicida* subsp. *salmonicida* strains from various regions of the world.

## Figures and Tables

**Table 2:** Correlation between the presence of the ASA_2927 to ASA_2933 genes and certain groups of *A. salmonicida* subsp. *salmonicida*.

Type of *AsaGEI*	Total Number of Strains Tested ^a^	TTSS Loss Sensitivity ^b^	Strains with ASA_2927 to ASA_2933 (%)
*AsaGEI1a*	20	++	100%
*AsaGEI2a* with prophage 3	16	-	0%
*AsaGEI2a* without prophage 3	8	+	0%
No *AsaGEI* and originating from Prov. of Quebec	11	-	0%
No *AsaGEI* and originating outside the Prov. of Quebec	10	+++	100%

^a^: The numbers indicated represent the sum of Table 1 and Table S3. ^b^: +++: 100% of the strains tested gave TTSS loss; ++: 50 to 99% of the strains tested gave TTSS loss; +: 1 to 49% of the strains tested gave TTSS loss; -: 0% of the strains tested gave TTSS loss.

## Data Availability

The data presented in this study are available in the article’s figures and tables and in the supplementary materials. The analyzed genome sequences are available on GenBank (NCBI). The raw data presented in this study are available on request from the corresponding author.

## Supplementary Materials

The following are available online at https://www.mdpi.com/2076-2607/9/1/85/s1, Table S1: Primers used in this study; Table S2: Genome sequences of *A. salmonicida* subsp. *salmonicida* used for the comparative genomic analysis; Table S3: Systematic analysis of the TTSS locus loss in Canadian strains of *A. salmonicida* subsp. *salmonicida* after incubation at 25 °C for two consecutive periods of 3 days. Table S4: Plasmids present in the strains used in this study.
